# Educational significance and challenges of conducting the objective structured clinical examination twice for midwifery students before and after clinical training: A longitudinal single university study during 2014–2019 in Japan

**DOI:** 10.1371/journal.pone.0278638

**Published:** 2022-12-01

**Authors:** Yuki Morikawa, Yukari Watanabe, Mayumi Yamauchi, Mayumi Yamamoto, Mamoru Morikawa, Kazumi Ishibiki, Mai Ohtomo, Michiko Miyazaki, Keiko Nakamura

**Affiliations:** 1 Graduate Program of Midwifery, Sapporo City University School of Nursing, Sapporo, Japan; 2 Department of Obstetrics and Gynecology, Hokkaido University Graduate School of Medicine, Sapporo, Japan; 3 Graduate School of Nursing, Sapporo City University School of Nursing, Sapporo, Japan; Tabriz University of Medical Sciences, ISLAMIC REPUBLIC OF IRAN

## Abstract

This study aimed to clarify the educational significance and issues associated with administering the objective structured clinical examination (OSCE) twice to midwifery students, i.e., before and after clinical training. In Sapporo City University in Japan, 37 assessment items of the OSCE were configured as “Overall,” with 17 items as midwifery’s normal delivery preparation (Part 1) and 20 items as midwifery’s normal delivery assistance (Part 2). All students had attended lectures with textbooks. The first and second OSCEs were conducted before and after the clinical training, respectively. The scores of 54 students were retrospectively analyzed over 6 years (2014–2019). The results of the first and second OSCEs were compared. Statistical analysis was performed using Mann–Whitney U test, Wilcoxon signed rank-sum test, Fisher’s exact test, and analysis of variance. The mean scores for “Overall” [0–37], “Part 1” [0–17], and “Part 2” [0–20] in the second OSCEs were significantly higher than those in the first OSCE (Overall: 22.7 vs 19.3, Part 1: 9.50 vs 7.71, Part 2: 13.2 vs 11.6, p<0.05, respectively). Regarding “Overall” and “Part 1,” a positive correlation was observed between the first and second OSCEs, wherein the full scores of “Part 1,” converted from 17 to 20 points to match the full scores of “Part 2,” were significantly lower than those of Part 2 (p<0.05, respectively). There was a positive correlation between the scores of the first and second OSCEs in “Part 1” and “Part 2” (p<0.05). The scores increased between the two OSCEs, and participants could objectively grasp the knowledge and skills. The OSCEs conducted twice were useful in skilling-up the normal delivery preparation and assistance skills of midwifery students. However, developing an advanced educational method might be necessary for the midwifery students’ preparation of normal delivery, because the scores in the OSCEs were lower.

## Introduction

The objective structured clinical examination (OSCE) is a competency-based assessment tool used globally in medical education. Harden et al (UK) reported the world’s first OSCE in 1975, which was designed to assess the clinical skills and competence of final year medical students while avoiding many drawbacks of traditional clinical examinations [1]. Following that, not only in medical education but also in nursing education and midwifery education, OSCE has been recognized as an evaluation method focusing on the psychomotor and emotional areas. OSCE also enables objective assessment of judgment, skills, manners, and so on required for clinical practice. Nursing OSCEs were extensively used across various nursing specialties in 33 countries in a previous review of 204 studies published between 1982 and 2018 and confirmed their validity, reliability, and acceptability in nursing education [2]. It has been reported that it could be a practical midwifery test for midwifery students who receive feedback on what they have practiced from simulated patients and teachers [3–8]. However, neither in Japan nor elsewhere, were there any reports comparing the midwifery OSCE results before and after the training or evaluating them over time.

Since its inception in 2010, the Graduate Program in Midwifery at Sapporo City University has adopted OSCE as a method of acquiring midwifery technical abilities and has incorporated it into education [9]. The goal is to clarify the achievement level and evaluation criteria of the technical content related to delivery assistance acquired during 1 year of the midwifery major as well as to systematize the objective evaluation method, which includes the psychomotor area and the emotional areas [9]. Since 2011, we have conducted the OSCE with the same content twice a year (June and February of the following year) before and after the clinical training (before the course is completed) while modifying the evaluation, and have arrived at the evaluation content of this study. In this research from 2014 to 2019, we will clarify the educational significance and issues of the OSCE based on the evaluation scores before and after clinical training on the OSCE during the delivery period in the education of the Graduate Program in Midwifery, Sapporo City University.

To the best of our knowledge, this is the first study worldwide to compare the midwifery OSCE results before and after clinical training for midwifery students. This study aimed to clarify the educational significance and issues associated with administering the OSCE twice to midwifery students, i.e., before and after clinical training.

## Materials and methods

### Study design

Retrospective longitudinal study.

### Setting

Graduate Program of Midwifery, Sapporo City University School of Nursing, Sapporo, Japan.

### Inclusion and exclusion criteria of survey targets

Every April, approximately 10 midwifery students with a nurse license enroll in the Graduate Program in Midwifery, Sapporo City University, with the goal of obtaining the qualifications for the National midwifery Examination, which is held in February of the following year. This study focuses on midwifery students enrolled in the one-year Graduate Program in Midwifery, at Sapporo City University between 2014 and 2019.

**Fig 1:**
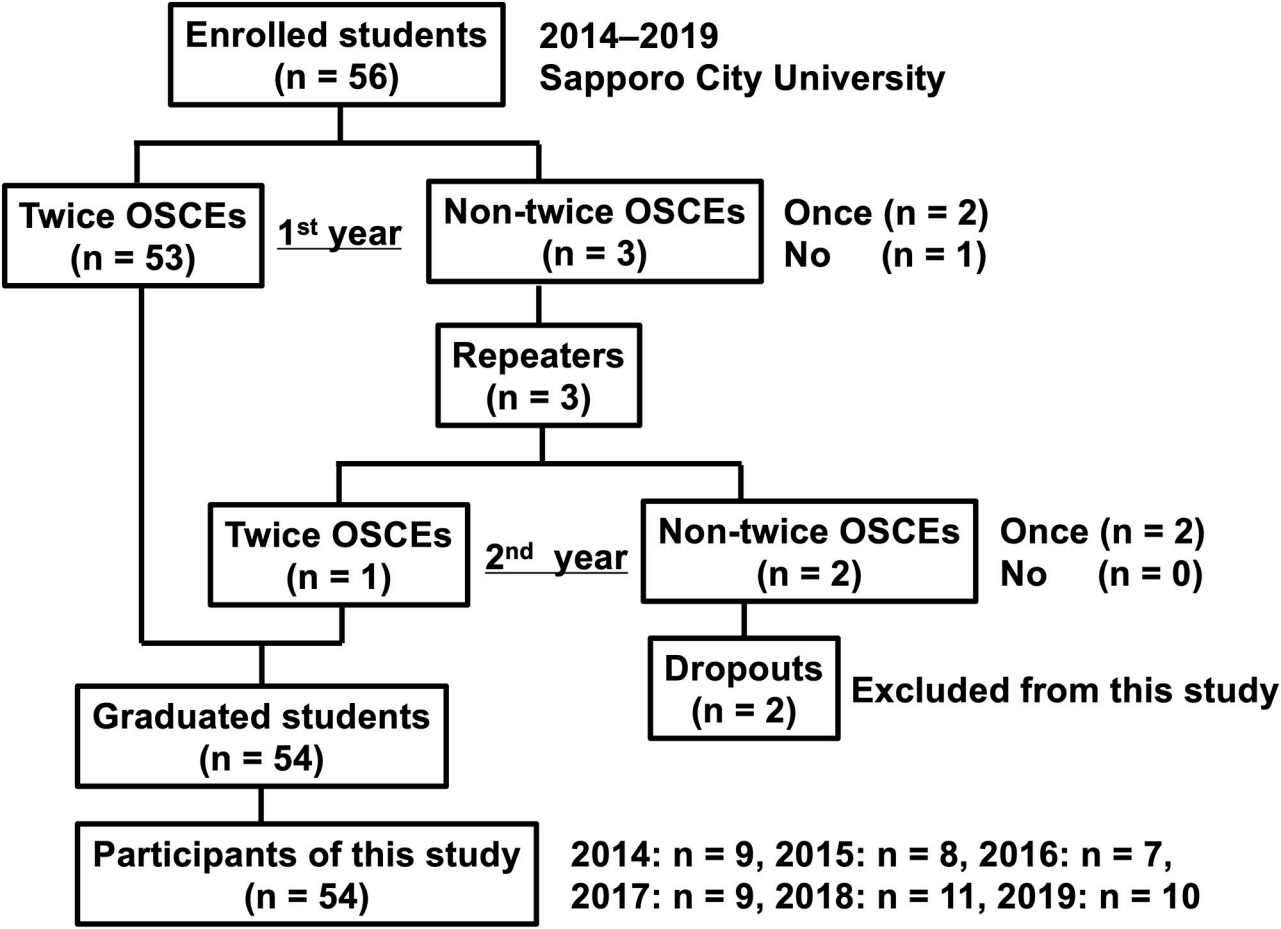
The flowchart of this study. OSCE, objective structured clinical examination.

In this study, only those participants without duplicate entries who had taken the OSCE twice, before and after clinical training, i.e., in June and February of the following year as per the target criteria were included. The target criteria were as follows: (1) students who have taken the OSCE twice without the holdovers (53 students); and (2) students who have repeated a year, with the grades for the second year of taking both exams before and after clinical training (1 student) (Fig 1).

In this study, students who took the exam only in June and dropped out are excluded (2 students) (Fig 1).

### Positioning of the OSCE

#### Positioning of the OSCE in the curriculum of the Graduate Program in Midwifery

The first OSCE, which will be held prior to the midwifery clinical training in June, will be conducted after the completion of “Delivery midwifery Diagnosis and Skill” (60 hours per 2 credits) of basic midwifery. As a prerequisite for advancing to midwifery clinical training, all students must pass the first OSCE in June. Furthermore, the second OSCE conducted before the completion of the advanced course in February of the following year is conducted in the “Exercise in midwifery (30 hours per 1 credit)” of integrated midwifery, and passing the second OSCE is a requirement for completing the advanced course.

#### Evaluation checklist and assignment sentence (scenario content) of OSCE

The OSCE evaluation checklist and the OSCE task statement were created when the OSCE was introduced into the curriculum in 2010. It has been revised since 2011, and it is now the current OSCE evaluation checklist and OSCE assignment sentence. The OSCE evaluation checklist for the delivery period is divided into two sections and contains a total of 37 evaluation items [9].

Table 1 shows the list of 37 items in the OSCE to educate midwifery students.

**Table 1:** The list of 37 items in the OSCE to educate midwifery students [9].

Overall: Part 1 + Part 2 (Q1–Q37)
**Part 1: Preparation for normal vaginal delivery (Q1–Q17)**
To assess the ability of midwifery students to prepare for normal vaginal delivery.
Q1. Can she place the instrument table and kick-buckets, considering work efficiency and safety?
Q2. Can she adjust the height of the delivery table?
Q3. Can she wear the operational gown according to the clean rules?
Q4. Can she wear the operational gloves according to the clean rules?
Q5. Can she conduct vulva disinfection according to the principle? (Can she maintain the Pean clean and the disinfection field with 1/2–2/3 of both intrafemoral measurements without the gap in the disinfection site?)
Q6. Can she prepare the clean fields according to the priority and maternal condition?
Q7. Can she prepare the items with the highest and higher priorities in order?
Q8. Can she connect the suction catheter cleanly?
Q9. Can she cover the tip of the suction catheter with clean gauze and fix it with forceps to keep it clean?
Q10. Can she arrange the items on the instrument table?
Q11. Can she support the breathing methods according to the maternal condition?
Q12. Can she properly protect the mother’s anus during labor pains?
Q13. Can she notice decelerations in fetal heart rates and encourage maternal deep breathing properly?
Q14. Can she turn and keep her eyes on the mother?
Q15. Can she explain the progress of delivery?
Q16. Can she encourage the mother?
Q17. Can she start to support the delivery cleanly? (Can she correctly use tweezers, change gloves when dirty, and maintain a clean field without maternal stool contamination?)
**Part 2: Assistance with normal vaginal delivery (Q18–Q37)**
To assess the ability of midwifery students to assist in normal vaginal delivery.
Q18. Can she apply her carpal with the cotton to protect the perineal at a position where the 1–2 cm of perineum could be seen without the gap from the mother’s anus?
Q19. Can she apply the entire of her left palm to the back of the infant’s head (the infant’s occipital region)?
Q20. Can she maintain the flexional state of the infant’s neck until the infant’s occipital nodules had slipped out under the mother’s pubic symphysis?
Q21. Can she simultaneously avoid the passage of both infants’ parietal nodules?
Q22. Can she prompt for maternal short breathing after the infant’s parietal nodules came out from the maternal pubic symphysis?
Q23. Can she compress the infant’s occipital nodules posterior-inferiorly and deliver the crown of the infant’s head?
Q24. Can she place her left hand on the infant’s head or forehead and pass the infant’s head, forehead, and face in order through the mother’s vulva slowly?
Q25. Can she wipe the infant’s face after the third rotation of delivery?
Q26. Can she confirm the presence of umbilical cord entanglements in the infant and cancel them?
Q27. Can she deliver 1/3–1/2 of the infant’s pre-existing shoulder after the fourth rotation of delivery, support the infant’s head with her left hand, and deliver the infant’s posterior shoulder while looking at the mother’s perineum?
Q28. Can she finish the maternal perineal protection, wipe the anus downwardly with the protective cotton ball, and throw the cotton ball away?
Q29. Can she grasp the infant safely? (Can she grip the infant’s upper arms and fix the infant’s back or grab the infant’s back through both infant’s armpits?)
Q30. Can she deliver the infant according to the mother’s pelvic lead line?
Q31. Can she confirm the birth time and infant’s gender and report to the mother?
Q32. Can she lay the infant down to avoid pulling the umbilical cord? (Can she check it with the fetal nursing staff?)
Q33. Can she perform the methods of supporting the infant’s first breath and appropriate suctioning of the infant?
Q34. Can she clean the infant’s whole body and take action to maintain the infant’s body temperature?
Q35. Can she judge and tell the infant’s Apgar score?
Q36. Can she appropriately ligate and amputate the umbilical cord? (Can she avoid pulling and squeezing of the umbilical cord? Can she clip the umbilical cord at 2–3 cm from the infant’s navel and grasp it using a few of Kochel at the additional 2–3 cm far? Can she cut the umbilical cord at an appropriate time using an appropriate method?)
Q37. Can she maintain a clean field and finish support the delivery? Can she wash her hands to maintain them clean and/or change her gloves?

OSCE: objective structured clinical examination.

“Part 1” has 17 evaluation contents about “preparation for normal vaginal delivery” and consists of 17 items that evaluate knowledge and skills. The goal of delivery preparation has been “to be able to prepare for delivery while keeping the mother and baby’s safety and comfort in mind.” The following are the action objectives: (1) Considering the priority, you can prepare a clean baby delivery area, (2) You can prepare the instruments in accordance with the priority, (3) You can teach the breathing method and the effort method based on the maternity woman’s condition, (4) You can make anal protection safe and comfortable, (5) You can prepare with consideration for safety, (6) You can observe the condition of the fetus, and take any necessary correspondence.

“Part 2” is 20 evaluation contents about “assistance of normal vaginal delivery” and consists of 20 items that assess knowledge and skills. The goal of delivery assistance is “to be able to provide delivery assistance while keeping the mother and baby’s safety and comfort in mind.” Action goals are as follows: (1) Can protect the perineum, (2) Can encourage short breathing, (3) Can assist the third rotation of the fetus, (4) Can assist the 4th rotation of the fetus, (5) Can confirm if there is an umbilical cord entanglement, (6) Can deliver the shoulder, (7) Can deliver the trunk, (8) Can correspond newborn babies immediately after birth.

The assessment is whether it is possible (1 point) or not (0 points). Part 1 has a maximum of 17 points, and Part 2 has a maximum of 20 points. The evaluation score is the average of the two teachers’ evaluations. The scenario for each task is determined by the evaluation contents of Part 1 and Part 2.

#### Methods of implementing the OSCE and the role of faculty members

At the venue, each station of Part 1 and Part 2 was set up, and the midwifery students moved in the order of Part 1 and Part 2, taking the OSCE at each station. Midwifery students read the assignment sentence in 1 minute and then provide midwifery care based on the simulated maternity condition (role of fetal progress) in 10 minutes. Midwifery students received feedback from the simulated maternity and the evaluator 3 minutes and 30 seconds after completion.

A delivery simulator (pelvic model) and a fetal simulator (fetal model) were used by one instructor in charge of simulated maternity (role of fetal progress) to advance the delivery scene according to the scenario. The two faculty members in charge of evaluating the student’s implementation checked 1 point or 0 points based on the evaluation criteria.

#### Evaluation staff of the OSCE

During this study period, six teaching staff members scored the students in the OSCE. On the other hand, the number of students scored by two out of six faculty members was small. As a result, the two faculty members were designated as evaluator E (and others). Evaluators A, B, C, and D were the other four faculty members.

#### Collection and analysis of data

In this study, all of the evaluation table results (37 items) were anonymized before being analyzed. Hence, subject characteristics such as the age (years), the presence/absence of service as a nurse, and the length of service as a nurse (years) of the students were not displayed.

We calculated the score of each item using the average of the score checked by two faculty members (1 or 0 points). Thus, all types of scores in each item were 1.0 (high score), 0.5, and 0.0 points (low score). The OSCEs had 37 items (“Overall”), including “Part 1” with 17 items and “Part 2” with 20 items. The possible range of scores of “Overall,” “Part 1,” and “Part 2” was 0.0–37.0, 0.0–17.0, and 0.0–20.0, respectively. The data are the results of the evaluation of students who took both the first (before the clinical training) and second OSCEs (after the clinical training).

#### Lecture, clinical training, and OSCE

All midwifery students had attended lectures on “delivery preparation” and “delivery assistance” with textbooks conducted by the evaluators (teachers) of Sapporo City University until the first OSCEs. All midwifery students had taken clinical training after the first OSCE and before the second OSCE. The clinical training was performed in some maternity hospitals outside Sapporo City University. Clinical training included “delivery preparation” and “delivery assistance” for 10 real pregnant women with some midwifery (staff of the maternity hospitals) and an evaluator (a teacher) of Sapporo City University.

### Outcomes

The primary outcome was to compare the OSCE scores before and after the clinical training. The secondary outcome was to compare the scores of delivery preparation (Part 1) and delivery assistance (Part 2).

Furthermore, additional outcomes were to compare the changes in OSCE scores over 6 years and the score difference between evaluators.

### Sample size

We calculated that this study needed 51 midwifery students, with a power of 0.8, an error level of 0.05, and an effect size of 0.5.

### Statistical analysis

All data are presented as median [minimum–maximum], or frequency. The statistical software JMP Pro, version 16.0 (SAS Institute Inc., Cary, NC, USA) was used for the statistical analysis. To compare means, Tukey–Kramer honestly significant difference tests or student’s *t*-tests were used. Medians were compared using the Mann–Whitney U test and Wilcoxon signed rank-sum test. To compare categorical variables, Fisher’s exact test was used. Pearson’s product-moment correlation coefficient was used to evaluate the linear correlation between the two variables. ANOVA was used to compare the trends over the course of 6 years in this study.

In all analyses, P < 0.05 was used to indicate statistical significance. *P*-values of <0.05 and correlation coefficient (*r*) of >0.25 or <−0.25 were used to indicate statistical significance for linear correlations.

This study failed to reveal the confounding variables according to the linear regression of data between OSCE scores before and after the clinical training and between OSCE scores of Part 1 and Part 2 (data not shown). Thus, this study had no confounding variables controlled in the statistical analyses and no confounders that were adjusted. While, we suspected the skill-up of evaluators in OSCE with the passing of the years and/or the difference of evaluators’ skill in OSCE might have the possibility of the confounding factors of OSCE scores. Thus, we analyzed the changes in OSCE scores over 6 years and the score difference between evaluators in OSCE in this study. This study had no missing data.

### Ethics statement

This study was carried out with the approval of Sapporo City University’s Institutional Review Board (No. 1213–1). Before participating in the research, all midwifery students were given informed consent by researchers other than the OSCE evaluator about the risk of participating in the research and provision of refusing to participate in the research. All participants provided written informed consent before participating in this study. The data of this study are released for public access via the website of the Graduate Program of Midwifery, Sapporo City University School of Nursing, according to the recommendations of the Ministry of Health, Labor, and Welfare (Japan).

## Results

During the survey period, 54 midwifery students (9 [7–11] per year) took the OSCE for six years (Fig 1).

### Scores of each evaluation item in the first OSCE (June) and second OSCE (February of the following year)

Table 2 displayed the scores of “Overall,” “Part 1,” and “Part 2” in the first OSCE (June) and second OSCE (February of the following year). The scores of “Overall” (37 points), “Part 1” (17 points), and “Part 2” (20 points) in the second OSCE were significantly higher than those in the first OSCE (Overall; 20.25 [6.0–30.0] vs. 23.5 [11.0–34.0], P = 0.0005, Part 1; 7.50 [1.5–14.0] vs. 9.5 [4.0–17.0], p = 0.0002, Part 2; 12.25 [3.5–19.5] vs. 13.75 [4.5–18.5], p = 0.0105).

**Table 2:** Scores of midwifery students in the first and second OSCEs.

	First OSCE (in June)	Second OSCE (in Feb^a^)	*P-value*
**Case number**	54 (100%)	54 (100%)	
**Overall: Part 1 + Part 2 (Q1–Q37, maximum score: 37 points)**	20.3 [6.0–30.0]	23.5 [11.0–34.0]	*0*.*0005*
**Part 1: Preparation for normal vaginal delivery (Q1–Q17, maximum score: 17 points)**	7.5 [1.5–14.0]	9.5 [4.0–17.0]	*0*.*0002*
**Part 2: Assistance with normal vaginal delivery (Q18–Q37, maximum score: 20 points)**	12.3 [3.5–19.5]	13.8 [4.5–18.5]	*0*.*0105*

All data are shown as median [minimum–maximum]. Statistical analysis was performed using Wilcoxon signed rank-sum test. OSCE: objective structured clinical examination, Feb^a^: February of the following year.

Tables 3 and 4 show the changes in the frequencies of students with each score (1.0, 0.5, and 0.0 points) in 37 items between the first and second OSCEs in Part 1 (Table 3) and Part 2 (Table 4). When the scores before and after the clinical training were compared, six items from Part 1 (Q2, Q6, Q7, Q9, Q13, and Q14) and four items from Part 2 (Q20, Q24, Q27, and Q29) were significantly higher after the clinical training than those before the clinical training. The comparison of scores before and after the clinical training revealed that one item from Part 1 (Q15) and one item from Part 2 (Q33) were significantly lower after the clinical training than those before the clinical training.

**Table 3:** Changes in the frequencies of students with each type of scores in 17 items of Part 1 (Q1–Q17) between the first and second OSCEs.

	First OSCE (in June), n = 54			Second OSCE (in Feb^a^), n = 54			*P-value*
	1.0 (high)	0.5	0.0 (low)	1.0 (high)	0.5	0.0 (low)	
**Q1**	33 (61.1%)	11 (20.4%)	10 (18.5%)	34 (63.0%)	13 (24.1%)	7 (13.0%)	0.7331
**Q2**	42 (77.8%)	6 (11.1%)	6 (11.1%)	51 (94.4%)	2 (3.7%)	1 (1.9%)	0.0397
**Q3**	21 (38.9%)	10 (18.5%)	23 (42.6%)	16 (29.6%)	9 (16.7%)	29 (53.7%)	0.5078
**Q4**	24 (44.4%)	12 (22.2%)	18 (33.3%)	26 (48.2%)	13 (24.1%)	15 (27.8%)	0.8789
**Q5**	10 (18.5%)	10 (18.5%)	34 (63.0%)	18 (33.3%)	8 (14.8%)	28 (51.9%)	0.2363
**Q6**	6 (11.1%)	10 (18.5%)	38 (70.4%)	25 (46.3%)	16 (29.6%)	13 (24.1%)	<0.0001
**Q7**	4 (7.4%)	12 (22.2%)	38 (70.4%)	23 (42.6%)	12 (22.2%)	19 (35.2%)	<0.0001
**Q8**	17 (31.5%)	3 (5.6%)	34 (63.0%)	21 (38.9%)	9 (16.7%)	24 (44.4%)	0.0826
**Q9**	11 (20.4%)	3 (5.6%)	40 (74.1%)	23 (42.6%)	6 (11.1%)	25 (46.3%)	0.0116
**Q10**	1 (1.9%)	4 (7.4%)	49 (90.7%)	5 (9.3%)	4 (7.4%)	45 (83.3%)	0.2985
**Q11**	17 (31.5%)	14 (25.9%)	23 (42.6%)	16 (29.6%)	21 (38.9%)	17 (31.5%)	0.3176
**Q12**	31 (57.4%)	18 (33.3%)	5 (9.3%)	37 (68.5%)	16 (29.6%)	1 (1.9%)	0.1846
**Q13**	18 (33.3%)	2 (3.7%)	34 (63.0%)	26 (48.2%)	7 (13.0%)	21 (38.9%)	0.0242
**Q14**	15 (27.8%)	23 (42.6%)	16 (29.6%)	32 (59.3%)	18 (33.3%)	4 (7.4%)	0.0008
**Q15**	37 (68.5%)	11 (20.4%)	6 (11.1%)	23 (42.6%)	19 (35.2%)	12 (22.2%)	0.0247
**Q16**	44 (81.5%)	6 (11.1%)	4 (7.4%)	36 (66.7%)	11 (20.4%)	7 (13.0%)	0.2262
**Q17**	4 (7.4%)	8 (14.8%)	42 (77.8%)	4 (7.4%)	10 (18.5%)	40(74.1%)	0.9418

All data are shown as frequencies (Overall frequencies of “1.0,” “0.5,” and “0.0” were 100% in the first and second OSCEs). Statistical analysis was performed using Fisher’s exact tests. OSCE: objective structured clinical examination. Feb^a^: February of the following year. Part 1 of OSCE: to assess the midwifery students’ ability to prepare for normal vaginal delivery.

**Table 4:** Changes in the frequencies of students with each type of score in 20 items of Part 2 (Q18–Q37) between the first and second OSCEs.

	1^st^ OSCE (in June), n = 54			2^nd^ OSCE (in Feb^a^), n = 54			*P-value*
	1.0 (high)	0.5	0.0 (low)	1.0 (high)	0.5	0.0 (low)	
**Q18**	12 (22.2%)	13 (24.1%)	29 (53.7%)	18 (33.3%)	12 (22.2%)	24 (44.4%)	0.4577
**Q19**	20 (37.0%)	16 (29.6%)	18 (33.3%)	27 (50.0%)	11 (20.4%)	16 (29.6%)	0.3661
**Q20**	19 (35.2%)	20 (37.0%)	15 (27.8%)	34 (63.0%)	9 (16.7%)	11 (20.4%)	0.0100
**Q21**	15 (27.8%)	15 (27.8%)	24 (44.4%)	18 (33.3%)	20 (37.0%)	16 (29.6%)	0.2797
**Q22**	31 (57.4%)	11 (20.4%)	12 (22.2%)	42 (77.8%)	5 (9.3%)	7 (13.0%)	0.0861
**Q23**	17 (31.5%)	15 (27.8%)	22 (40.7%)	26 (48.2%)	16 (29.6%)	12 (22.2%)	0.0849
**Q24**	16 (29.6%)	14 (25.9%)	24 (44.4%)	28 (51.9%)	14 (25.9%)	12 (22.2%)	0.0289
**Q25**	41 (75.9%)	6 (11.1%)	7 (13.0%)	49 (90.7%)	2 (3.7%)	3 (5.6%)	0.1454
**Q26**	30 (55.6%)	12 (22.2%)	12 (22.2%)	40 (74.1%)	9 (16.7%)	5 (9.3%)	0.1048
**Q27**	8 (14.8%)	12 (22.2%)	34 (63.0%)	15 (27.8%)	18 (33.3%)	21 (38.9%)	0.0469
**Q28**	30 (55.6%)	12 (22.2%)	12 (22.2%)	30 (55.6%)	13 (24.1%)	11 (20.4%)	1.000
**Q29**	14 (25.9%)	14 (25.9%)	26 (48.2%)	22 (40.7%)	24 (44.4%)	8 (1.8%)	0.0009
**Q30**	30 (55.6%)	14 (25.9%)	10 (18.5%)	31 (57.4%)	12 (22.2%)	11 (20.4%)	0.9313
**Q31**	42 (77.8%)	9 (16.7%)	3 (5.6%)	37 (68.5%)	12 (22.2%)	5 (9.3%)	0.5707
**Q32**	25 (45.5%)	10 (18.5%)	19 (35.2%)	30 (55.6%)	12 (22.2%)	12 822.2%)	0.3427
**Q33**	27 (50.0%)	12 (22.2%)	15 (27.8%)	16 (29.6%)	10 (18.5%)	28 (51.9%)	0.0311
**Q34**	32 (59.3%)	12 (22.2%)	10 (18.5%)	39 (72,2%)	9 (16.7%)	6 (11.1%)	0.3945
**Q35**	41 (75.9%)	6 (11.1%)	7 (13.0%)	39 (72.2%)	4 (7.4%)	11 (20.4%)	0.5535
**Q36**	21 (38.9%)	15 (27.8%)	18 (33.3%)	19 (35.2%)	13 (24.1%)	22 (40.7%)	0.8047
**Q37**	32 (59.3%)	10 (18.5%)	12 (22.2%)	31 (57.4%)	16 (29.6%)	7 (13.0%)	0.2814

All data are shown as frequencies (Overall frequencies of “1.0,” “0.5,” and “0.0” were 100% in the first and second OSCEs). Statistical analysis was performed using Fisher’s exact tests. OSCE: objective structured clinical examination. Feb^a^: February of the following year. Part 2 of OSCE: to assess midwifery students’ ability to assist normal vaginal delivery.

### Changes in the scores of each student

**Fig 2:**
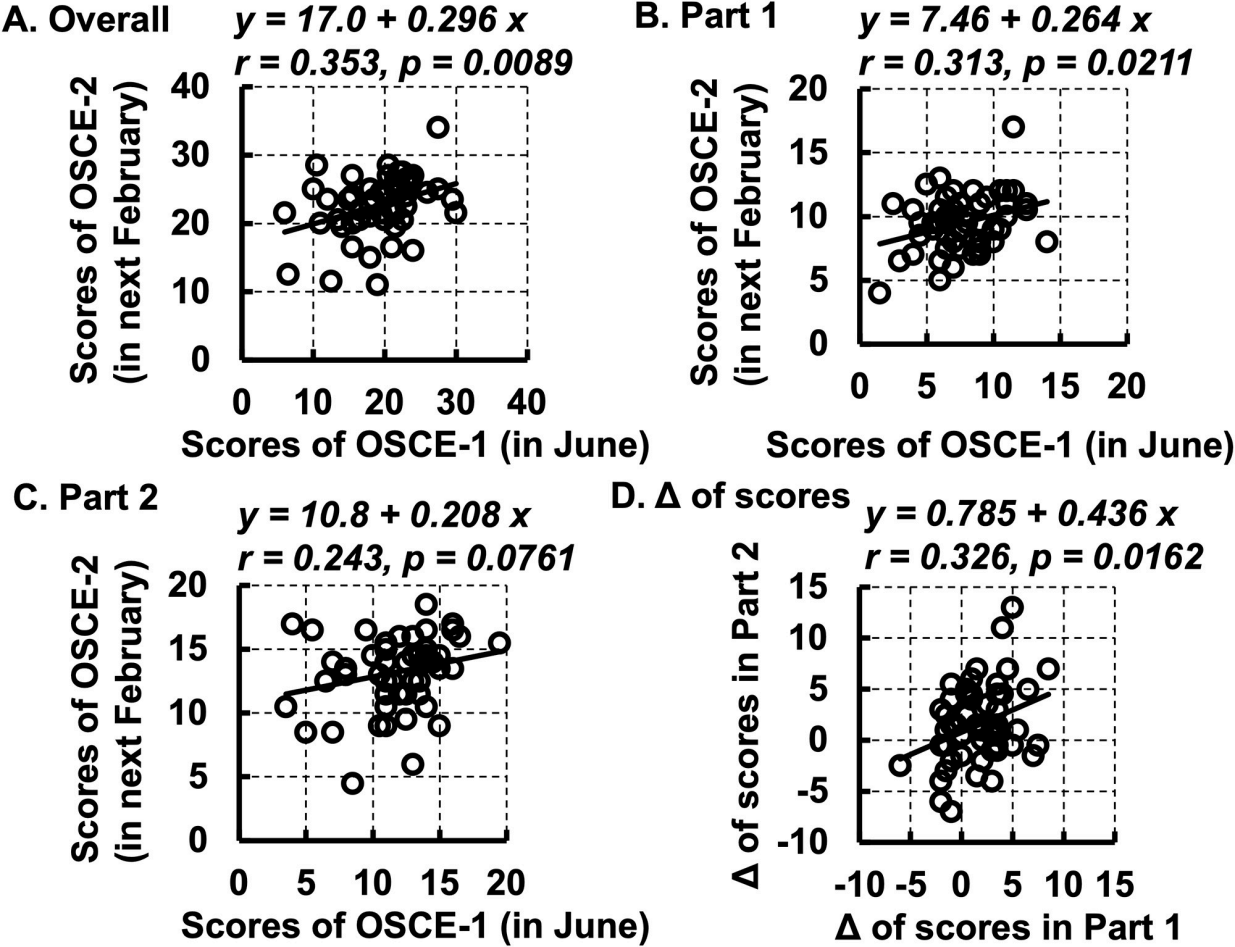
Correlations of scores of the first (in June) and second OSCEs (in February of the following year) and the increase in scores from the first to the second OSCE in Part 1 and Part 2. A. Scores of the first and second OSCEs in Overall (Part 1 + Part 2). B. Scores of the first and second OSCEs in Part 1. C. Scores of the first and second OSCEs in Part 2. D. Increase in scores from the first OSCE to the second OSCE in Part 1 and Part 2. OSCE: objective structured clinical examination. Part 1 of OSCE: to assess the midwifery students’ ability to prepare for normal vaginal delivery. Part 2 of OSCE: to assess midwifery students’ ability to assist normal vaginal delivery.

Plots were created to display each student’s Overall and Part 1 scores. A positive correlation was observed between the first and second OSCEs (p = 0.0089, p = 0.0211) (Fig 2A and 2B). However, the score of Part 1 was not correlated between the first and second OSCEs (Fig 2C). The increased score, which is the change in the median score from the first OSCE to the second OSCE (an increase of score = the second OSCE’s score − the first OSCE’s score), in Overall, Part 1, and Part 2 were 3.5 (−8.5–18.0), 2.0 (−6.0–8.5), and 1.0 (−7.0–13.0), respectively. The frequency of negative increase in scores among the 54 midwifery students was 22.2%, 27.8%, and 35.2% for Overall, Part 1, and Part 2, respectively. Conversely, the increase in score revealed a positive correlation between Part 1 and Part 2 (p = 0.0162) (Fig 2D).

### Scores of Part 1 and Part 2 in the first and second OSCEs

The results of the first and second OSCEs were compared for Part 1 and Part 2. The median (minimum–maximum) of Part 1 was calculated after converting to 20 points and compared with the median value of Part 2. Part 1 was significantly lower in both the first and second OSCEs (first OSCE: Part 1: 8.82 (1.8–16.5) and Part 2: 12.25 (3.4–19.5), p < 0.0001; second OSCE: Part 1: 11.18 (4.7–15.3) and Part 2: 13.75 (4.5–18.5), p = 0.0073).

### Changes in OSCE scores over 6 years

Table 5 presents the changes in the median scores of the first and second OSCEs over 6 years. The improvement in the scores over 6 years in the first OSCE showed a significant upward trend in all scores of “Overall” (p = 0.0003), “Part 1” (p < 0.0001), and “Part 2” (p = 0.0042), as determined using ANOVA. The scores of “Overall” and “Part 1” showed a significant upward trend in the second OSCE (p = 0.0099 and p = 0.0162, respectively), as determined using ANOVA. However, the second OSCE showed no upward trend in “Part 1.” The scores for “Overall,” “Part 1,” and “Part 2” were the lowest in 2015 in the first OSCE. Conversely, the scores for “Overall,” “Part 1,” and “Part 2” were the lowest in 2014 in the second OSCE.

**Table 5:** The trend of score in our OSCE to educate midwifery students.

	Overall	2014 ^A^	2015 ^B^	2016 ^C^	2017 ^D^	2018 ^E^	2019 ^F^	P-value <0.05
Number of cases	54	9	8	7	9	11	10	
**First OSCE (in June)**								
**Overall**	20.25 [6.0–30.0]	15.5 [12.5–24.0]	11.5 [6.0–23.0]	20.0 [16.5–22.0]	21.5 [16.5–24.0]	21.0 [19.5–30.0]	22.25 [10.5–27.5]	B vs. C, B vs. D, B vs. E, B vs. F, E vs. A, E vs. C
**Part 1** ^a^	7.5 [1.5–14.0]	6.0 [4.0–9.0]	4.25 [1.5–9.0]	7.0 [5.5–10.0]	7.0 [5.0–11.5]	9.0 [6.5–14.0]	10.75 [6.5–12.5]	B vs. C, B vs. D, B vs. E, B vs. F, E vs. A, E vs. D, F vs. A, F vs. C, F vs. D
**Part 2** ^b^	12.25 [3.5–19.5]	11.0 [7.0–15.0]	8.0 [3.5–14.0]	12.5 [8.0–14.5]	13.0 [10.5–16.5]	13.0 [11.0–19.5]	11.0 [4.0–15.0]	B vs. C, B vs. D, B vs. E, E vs. A, E vs. F
**Second OSCE (in Feb** ^c^ **)**								
**Overall**	23.5 [11.0–34.0]	19.5 [11.0–24.0]	22.0 [12.5–27.0]	24.5 [20.5–26.0]	22.0 [15.0–27.5]	24.5 [16.5–34.0]	24.75 [20.5–28.5]	A vs. C, A vs. D, A vs. E, A vs. F
**Part 1**	9.5 [4.0–17.0]	8.0 [5.0–10.0]	9.25 [4.0–11.0]	9.5 [7.5–12.0]	10.0 [6.0–13.0]	9.5 [7.0–17.0]	10.75 [8.0–12.0]	NS
**Part 2**	13.75 [4.5–18.5]	10.5 [4.5–14.5]	13.25 [8.5–16.5]	14.0 [11.5–16.0]	12.5 [9.0–16.5]	14.5 [9.5–18.5]	14.0 [11.5–17.0]	A vs. C, A vs. E, A vs. F
**Increase in the OSCE scores**								
**Overall**	3.5 [−8.5–18.0]	2.0 [−8.0–8.5]	10.25 [−0.5–15.5]	4.5 [2.5–5.0]	3.0 [−3.0–7.0]	3.0 [−8.5–8.0]	2.5 [−2.5–18.0]	A vs. B, B vs. D, B vs. E
**Part 1**	2.0 [−6.0–8.5]	3.0 [−2.0–5.0]	3.75 [−1.5–8.5]	3.5 [−1.0–5.0]	2.0 [−1.0–7.5]	0.5 [−6.0–5.5]	0.5 [−2.0–5.0]	NS
**Part 2**	1.0 [−7.0–13.0]	−0.5 [−7.0–5.0]	5.25 [−2.0–11.0]	1.0 [−0.5–5.5]	−0.5 [−3.5–4.5]	1.5 [−4.0–4.5]	3.25 [−1.5–13.0]	A vs. B, B vs. D, B vs. E

All data are shown as median [minimum–maximum].

Statistical analysis was performed using ANOVA.

OSCE; objective structured clinical examination. NS is not significant.

^a^ Part 1; OSCE test to assess the midwifery students’ ability to prepare for normal vaginal delivery.

^b^ Part 2; OSCE test to assess midwifery students’ ability to assistant normal vaginal delivery during.

^c^ in Feb; February of the following year.

### Score difference between evaluators

The evaluation of Part 1 and Part 2 of each OSCE was conducted by the same evaluator. During the time of this study, the number of people in charge of evaluation in the 1^st^ OSCE was 27 for A, 37 for B, 16 for C, 9 for D, and 19 for E. The number of people in charge of evaluation in the 2^nd^ OSCE, was 31 for A, 41 for B, 13 for C, 9 for D, and 14 for E. The number of people in charge of evaluators A–E did not differ significantly (p = 0.8210).

In terms of evaluators’ scores, a specific evaluator recognized a significantly high score in the Overall and Part 1 of the 1^st^ OSCE and the 2^nd^ OSCE respectively (p < 0.0001). However, there was no significant difference in Part 2 between the 1^st^ OSCE and the 2^nd^ OSCE.

## Discussion

The following four points were clarified in this study. (1) Both before and after clinical training, the Part 1 score was significantly lower than that of Part 2. Furthermore, Part 1 failed to meet the achievement criteria. (2) When compared to before the clinical training, the score for Overall, Part 1, and Part 2 increased significantly after the clinical training. (3) The 6-year annual change in the evaluation score showed a significant upward trend. (4) In terms of evaluator evaluation scores, a significantly high score was found in Part 1 by a specific evaluator.

This is the first study that targeted midwifery students and used the same OSCE checklist both before and after clinical training to clarify the characteristics of the difference between the two OSCEs scores and the OSCE’s longitudinal tendency. Large individual differences in the acquisition of skills among midwifery students were found in this study. Midwifery students met 60% of the evaluation criteria for the practical ability of delivery assistance both before and after clinical training, but delivery preparation is inadequate. As a result, it was suggested that more education be considered to further improve skills in midwifery practice, particularly strengthening individual guidance for midwifery students with low technical abilities.

In this study, as a method of acquiring midwifery technical ability, OSCE regarding technical ability during delivery was carried out before and after the clinical training using the same checklist. As a result, the technical ability after clinical training was significantly higher than that before the clinical training, and the achievement status has reached 60% of the passing standard. Hence, clinical training may provide an opportunity to improve technical ability. In educational institutions, the OSCE for midwifery students was still rarely used. Previous studies only used it as an education and evaluation tool to prepare for clinical training, or as a confirmation of the achievement of practical ability before employment [4, 6, 10]. This was the first study to evaluate the students’ achievement status by conducting the OSCE longitudinally using the same checklist. The educational benefit of using the same implementation content and checklist would be the ability to objectively grasp the degree of achievement of technical ability in vaginal delivery before and after the clinical training. The OSCE results prior to clinical training were formative assessments, which clarified the tasks for the training and motivated them to acquire skills. Furthermore, understanding the results of the OSCE after the clinical training (before completion) would clarify the issues for employment preparation and would lead to motivation to continuously acquire knowledge and skills.

There were studies on the assistance of delivery and the third period of delivery in our search for previous studies [11]. However, we were unable to locate any studies that divided normal delivery into preparation for normal delivery (Part 1) and assistance for normal delivery (Part 2) and reported them in detail. This time, by dividing the OSCE into two stations, preparation of normal delivery (Part 1) and assistance of normal delivery (Part 2), it was clear that preparation of normal delivery (Part 1) was significantly lower than the assistance of normal delivery (Part 2) in both before and after the clinical training. Furthermore, it was also clarified that, while delivery preparation after the clinical training was significantly improved compared to before the clinical training, it did not meet 60% of the acceptance criteria. Because the time devoted to the practice for preparation of delivery (Part 1) prior to the OSCE before clinical training was limited, the delivery preparation score was deemed low. Furthermore, it could be argued that the motivation for normal delivery preparation (Part 1) was insufficient when compared to normal delivery assistance (Part 2) in the lectures and exercises “Delivery midwifery Diagnosis and Skill.”

In Japan, there is a background of integration and a decrease in obstetric facilities, a decrease in the number of births, and an increase in high-risk delivery. In addition, midwifery students are required to complete the number of delivery training which are designated by the rules during the limited clinical training period. Therefore, the midwifery students surveyed had to complete maternity training at three clinical facilities. As a result of differences in delivery preparation procedures and methods, midwifery students who received practical training at multiple facilities might find it difficult to learn the generalized delivery preparation method known as OSCE which might have resulted in a low score after clinical training.

The achievements of “preparation of normal delivery (Part 1)” and “assistance of normal delivery (Part 2)” were correlated with each midwifery students in this study. In other words, while the speed and degree of improvement of abilities vary depending on the individual midwife student, they may be increasing the abilities required for both preparations of “normal delivery (Part 1)” and “assistance of normal delivery (Part 2)” by repeating opportunities for clinical training during the delivery period.

Concerning the achievement status of each evaluation item, consideration for the environment for safe delivery assistance and skill acquisition for environment preparation are both low. Items related to “knowledge” and “skills and actions” are listed as essential competencies for midwifery practice by the International Confederation of Midwives. Some of the items are “Providing information, support, and encouragement to women and supporters,” “Providing polite one-on-one care,” “Proposing and supporting women to use methods to deal with labor pains,” and “Ensuring a clean environment, as well as clean equipment, and heating” [12]. As a result, it is desirable for the items related to delivery preparation to meet the passing criteria at the time of completion in order to improve maternal care, and OSCE evaluation of technical ability is meaningful. On the other hand, “Explanation for the progress of delivery (to the maternity women)” decreased significantly after the clinical training. It is not possible to concentrate solely on delivery preparation (securing a clean field and setting equipment) because the response to the progressing delivery is also performed at the same time. Hence, learning to prepare for delivery will take time for midwifery students. In addition, it will take time to master the “Explanation of the labor progress (to the mother)” that is performed concurrently with the preparation of delivery. Additional training programs to improve their skill may be required.

The “Breathing assistance and suction method for infants” follows the neonatal cardio-pulmonary resuscitation algorithm, which is based on the Consensus on Science with Treatment Recommendations and provides respiratory assistance for newborns immediately after birth. As a result, in the case of normal delivery assistance, there may be few opportunities to provide the assistance. However, because breathing assistance is an urgent and important skill, it was suggested that training and motivation be provided to ensure that breathing assistance and suction skill can be performed at any time and under any circumstances.

Despite the presence or absence of a significant difference among “Overall,” “Part 1,” and “Part 2,” the evaluation scores before the clinical training are increasing almost every year. After the clinical training, “Overall” and “Part 2” showed a significant difference from the year when the study began, and it increased almost every year after that. There were two reports on the annual transition of the evaluation results, a 4-year study by doctors [13] and a 5-year study by pharmacists [14], but neither showed an upward trend. It is claimed that the results of the OSCE can clarify the weaknesses of students and provide opportunities for improving teaching methods [1], and this result would imply that the teaching method may have been improved.

To avoid evaluation bias caused by only one evaluator, the midwifery OSCE assigned two evaluators to one student. Because it has been pointed out that evaluation by a two-person system is inconsistent, evaluation criteria have been established [15]. There was a significant difference in the scores of each evaluator in this study. The following biases can be considered as factors. There was a bias in the evaluation ability of teachers in the evaluation results because the evaluation teachers were different for each year, as well as visual inconsistency due to the evaluator’s standing position, and ambiguity of memory due to continuous technical confirmation. The professional background of the evaluator influences the difference in the evaluation score, according to an OSCE research report on veterinary education [16]. The evaluator of this OSCE was a faculty member with a midwifery qualification, but it might have been influenced by the number of years of service as a midwife and the number of cases for assistance of delivery. Also, there was no significant difference in the report on the difference between experienced and inexperienced evaluators for students with excellent grades, but when evaluating borderline students, inexperienced evaluators were reported to score severely [17], and this may be related to the number of years of teaching experience and the number of years of OSCE evaluation work.

Differences between evaluators have an impact on the reliability and impartiality of the OSCE, which objectively assesses clinical performance. In the future, before each OSCE, we will conduct a mock practice so that the evaluation criteria among teachers match. We will review the video shot in accordance with the evaluation criteria of each item at the end of each OSCE, share questions and unclear points after the OSCE, and hold demonstrations when new evaluators join. We will hold careful meetings and make every effort to match the evaluations. Furthermore, calculating the match rate of each evaluation item, examining the evaluation criteria for items with a large difference will be examined.

This study also had two limitations. First, the number of midwifery students was small. we will be able to conduct the multicenter study in the future. However, the multicenter study may have the bias of data among the universities (Graduate Program of midwifery), because each university will have its methods of normal delivery preparation and assistance. As a result, without the multitude of enrolled students at our university, it may be difficult to increase the number of midwifery students in future studies. Second, it was a retrospective cohort study without a control group of midwifery students (who had undergone the 2^nd^ OSCE after clinical training alone). Further research, such as prospective case-control studies or prospective randomized trials, may be required.

The technique of human vaginal delivery is universal; thus, almost all graduate programs of midwifery and the graduate school of nursing in the university can use our scoring system and our list of items to score OSCEs for midwifery students (Table 1). However, this study had a limitation in terms of external validity because some universities (especially in developing countries) might not have a delivery simulator (pelvic model) and a fetal simulator (fetal model). Meanwhile, all 37 items of the OSCE in this study could be modified to conform to the conditions of each university without a delivery simulator and a fetal simulator. Furthermore, the practice of conducting the OSCE twice would have applicability not only in the education of midwifery students but also in the education of nursing students and medical students.

Concerning vaginal delivery preparation, it would be preferable to consider educational methods and motivate them from the standpoints of safety and comfort. furthermore, it was clarified that technical ability for vaginal delivery could be improved by accumulating practical experience, so the method of continuing education after completion should be considered.

The evaluation is carried out by two faculty members, but in order to ensure reliability and validity, it may be necessary to create detailed evaluation criteria at a level that makes no difference depending on the evaluators and to increase the match rate.

## Conclusion

Implementing the twice-yearly OSCE in the one-year midwifery basic education course allows for an objective assessment of knowledge and technical acquisition progress. It will lead to task clarification for midwifery students prior to practical training and employment. Teachers (evaluators) could get feedback on their teaching methods.

The improvement in the achievement status for delivery preparation is lower than for delivery assistance, and the acceptance criteria have not been met even before completion. As a result, motivation for learning and educational methods for delivery preparation is required.

## Supporting information

S1 File Women with OSCE data. Dataset.
(XLSX)
